# A Comparative Analysis of Mast Cell Quantification in Five Common Dermatoses: Lichen Simplex Chronicus, Psoriasis, Lichen Planus, Lupus, and Insect Bite/Allergic Contact Dermatitis/Nummular Dermatitis

**DOI:** 10.5402/2012/759630

**Published:** 2012-02-01

**Authors:** Nikhil Patel, Amir Mohammadi, Ronald Rhatigan

**Affiliations:** Department of Pathology and Laboratory Medicine, University of Florida College of Medicine-Jacksonville, Jacksonville, FL 32209, USA

## Abstract

There is a large body of literature demonstrating an important role of mast cells in adaptive and innate immunity. The distribution of mast cells in the skin varies in different parts of the body. It is well known that mast cells are important for effector functions of classic IgE-associated allergic disorders as well as in host defense against infective agents and influence the manifestation of autoimmune diseases. We aimed to quantify mast cells in five common dermatoses and compare them statistically with respect to the immunostains. We retrieved paraffin-embedded tissue sections from the archives of the Pathology Department at the UF, Jacksonville, for five cases with each of the above diagnosis from the last three years. We performed CD-117 and tolidine blue stains on each one of them. The presence or absence of mast cells was evaluated and quantified. We observed that, in the skin, mast cells are mainly located close to the vessels, smooth muscle cells, hair follicles, and nerve ending. Our study showed that the mast cell distribution pattern is different across the two methods of staining for the five aforesaid dermatoses. The other important observation was the dendritic morphology of the mast cells.

## 1. Background

Dermatoses are a broad term, which includes any skin diseases, which are not characterized by neoplasm. Mast cells were first described based on staining of cytoplasmic granules, by Ehrlich in 1877 [1]. There is a large body of literature indicating an important role of mast cells in adaptive and innate immunity. The distribution of mast cells in the skin varies in different parts of the body. They are usually in higher number at the extremities and lower at the trunk [2]. Within the skin, the number of mast cells is 10-fold in the upper dermis as compared to the subcutis. However, there is no difference between gender and age groups [3]. It is well known that mast cells are important for effector functions of classic IgE-associated allergic disorders as well as in host defense against parasites, viruses, and bacteria and also influence the manifestation of autoimmune diseases including psoriasis, rheumatoid arthritis (RA), or bullous pemphigoid (BP). In the skin mast cells are mainly located close to the vessels, smooth muscle cells, hair follicles, and nerve ending [3]. Mast cells are derived from hematopoietic progenitor cells and mature in the local tissue where they reside. Once they mature in the tissues, they are associated with a partly tissue-specific pattern of mediators in their granules. Based on types of proteinases, human mast cells can be divided into mainly tryptase- and -chymase- (MCTC-), tryptase- (MCT-), or chymase- (MCC-) containing subtypes. MCTC mast cell type predominates in the dermis of the skin and submucosa, whereas MCT mast cell type predominates in the lung and bowel submucosa [4, 5]. The mast cell number, distribution, and functions change in different subtypes of dermatoses. There is not much published data regarding the analysis and quantification of mast cells in the five most common dermatoses, that is, lichen simplex chronicus, Psoriasis, lichen planus, lupus, and insect bite/allergic contact dermatitis/nummular dermatitis. Since it is usually not possible to differentiate insect bite, allergic contact dermatitis, and nummular dermatitis based on histology, we have combined them together in one category.

## 2. Materials and Method

We retrieved formalin fixed-paraffin-embedded tissue sections from the archives of the pathology department at the UF Shands Jacksonville for five cases with each of the above diagnosis from the last three years (2008 to 2011). These had been processed for routine histology using standard protocols. Briefly, they were fixed in 5% buffered formalin, and the sections were stained with hematoxylin and eosin dyes (H&E), CD-117 (C-kit), and Toluidine blue. Formalin was used as a fixative because formalin-fixed tissue yields the same results of mast cells compared to Carnoy's fluid. The cases were chosen based on the diagnosis made earlier, using the Co-path software. We performed both CD-117 (C-Kit) and toluidine blue stains on each one of them. The presence or absence of mast cells was evaluated by two pathologists at 40x magnification and when present was quantified. Several fields (minimum 5 fields) of 40x magnification were evaluated, and a mean was determined for each of the two staining methods. We did a comparative analysis for mast cell quantification across these different diagnoses.

## 3. Statistical Analysis

Due to small number of cases in each category of diseases, we used a nonparametric test, the Mann-Whitney *U* test (and reported the medians together with 25th and 75th percentile) to determine whether there was a significant difference between the staining methods (CD-117 (C-kit) v/s toluidine blue) for revealing the mast cells. We considered a *P* value of <0.05 to be statistically significant.

## 4. Results

There were a total of 25 patients in the study (15 females and 10 males). The distribution of gender was variable across all the disease entities except lupus, where all the patients were female (see Table 1). Also, in the category of lichen simplex chronicus, we had to eliminate one patient (male) as the CD-117 (C-kit) and toluidine blue stains were not adequate for interpretation due to minimal dermal tissue. The mean age of subjects plus/minus standard deviation was 37.2 ± 11.8 for psoriasis, 48.3 ± 10.9 for lichen simplex chronicus, 47.2 ± 15.5 for lupus, 51.6 ± 13.6 for lichen planus, and 19 ± 11.2  for insect bite/allergic contact dermatitis/nummular dermatitis, respectively. The mean age across all these disease entities was 46.6 ± 12.7 (see Table 1). According to the published literature, lichen simplex chronicus, lichen planus, or lupus is observed more commonly in females compared to males while psoriasis affects men and women equally. There is poor epidemiologic data regarding insect bite/allergic contact dermatitis/nummular dermatitis. Our study somewhat supports this observation except for lichen planus; however, the sample size is small to comment on this. Results are expressed as median. Overall, the distribution of mast cells is different across the two methods (*P* < .0001) (see Table 2). The median number of mast cells revealed using CD-117 (C-Kit) method was 17.5 compared to the median number of mast cells revealed using toluidine blue method which was 3.8. There was no statistical significance between the two methods for lichen simplex chronicus (*P* = 0.110). With the other disease entities, the *P* value was statistically significant. (psoriasis: *P*  value ≤ 0.0012, lupus: *P*  value = 0.024, lichen planus: *P*  value = 0/036, insect bite/allergic contact dermatitis/nummular dermatitis: *P*  value = 0.009) distribution (see Figure 2).

### 4.1. Histological Examination

#### 4.1.1. Morphology

**Figure 1:**
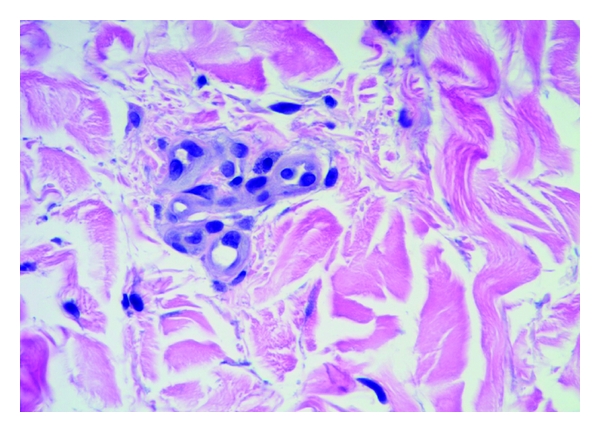
Psoriasis with mast cells next to the capillary vessel. H&E, 40x.

**Figure 2:**
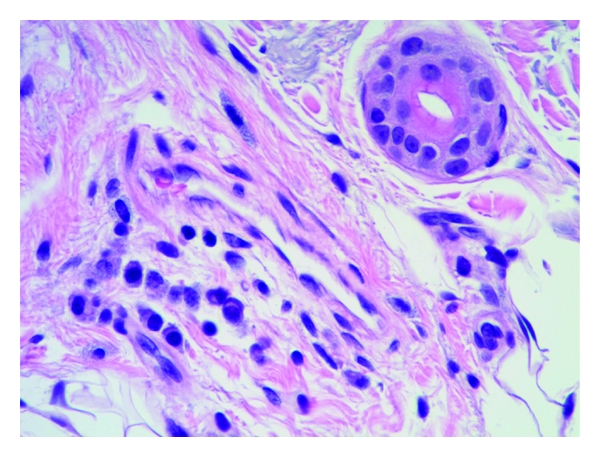
Lichen simplex showing mast cells in the vicinity of a capillary vessel. H&E, 40x.

**Figure 3:**
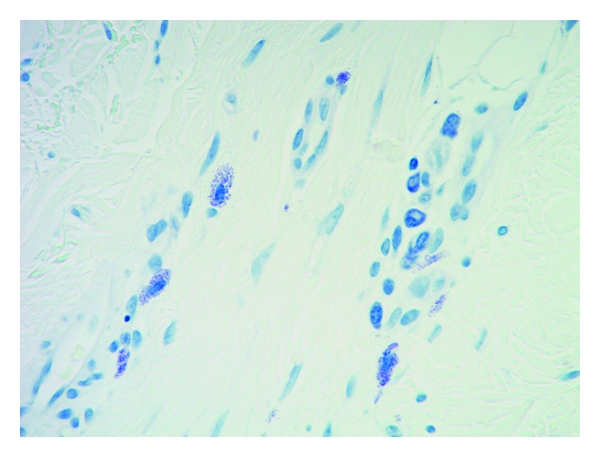
Mast cells stained with toluidine blue showing intracytoplasmic granules 40x.

**Figure 4:**
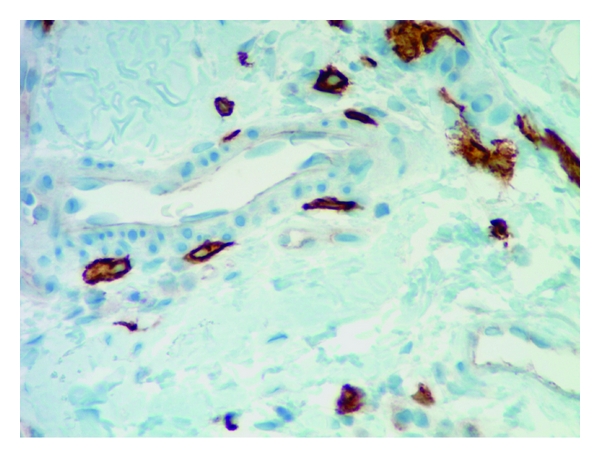
Mast cells stained with C-kit (CD117) next to the vessel 40x.

Mast cells were found throughout the dermis and were concentrated in the vicinity of vessels and skin appendages (Figures 1 and 2). They had a blue rounded nucleus, and their granules were metachromatically stained reddish purple with toluidine blue and brown with CD-117 (C-kit) stain (Figures 3 and 4). No mast cells were seen in the epidermis. C-kit (CD 117) is expressed by hematopoietic stem cells and is maintained during myeloid differentiation. It is thus an early marker for mast cell precursors. Mast cells continue to express CD-117 (C-kit) throughout the lifetime of the cell, unlike most other cells (including basophils), which lose this marker during their development.

#### 4.1.2. Evaluation of Fixative and Staining

Several studies have been done evaluating the fixative used for histology. Fixation in formaldehyde is superior to Carnoy's fixative as per Damsgaard TE and colleagues [6]. We used 2 methods of stain, that is, CD-117 (C-kit) and toluidine blue. The two methods reveal different distribution with a *P* value of less than 0.0001

#### 4.1.3. Quantification of Mast Cells

The number of mast cells stained with CD-117 (C-kit) and toluidine blue stains in biopsies from patients is shown in Table 2. A manual count by two pathologists under 40x magnification was performed. There were greater numbers of mast cells in lesional skin which were stained by CD-117 (C-kit) compared to the toluidine blue. The *P* value was significant across all the disease entities using CD-117 (C-kit) and toluidine blue stain except Lichen simplex chronicus where there was no significant difference in the two stains for mast cell evaluation.

## 5. Discussion

Mast cells are well known as effector cells of IgE-mediated allergic reactions. The important functions of mast cells in different diseases including innate immunity and the induction and regulation of adaptive immune responses have been reported [7–11]; however, their role in pathogenesis of the dermatological diseases is not fully understood. There is evidence of increase in the number of mast cells with degranulation in contact dermatitis. It is suggested that through the production of mediators, cytokines, and chemokines, they contribute in the pathogenesis of the diseases. We decided to quantify the mast cells to understand their significance and role as the effector in these 5 cutaneous diseases.

Mast cells are identifiable on H&E staining, but because of compar able morphology with other mononuclear cells, such as monocytes, histiocytes, lymphocytes, and nevomelanocytes, they may require special staining for verification. Different staining like Giemsa, toluidine blue, and Leder stain have been used, but immunohistochemistry is now often employed using C-kit (CD 117). We observed that, in the skin, mast cells are mainly located close to the vessels, smooth muscle cells, hair follicles, and nerve ending similar to the previously published data.

**Figure 5:**
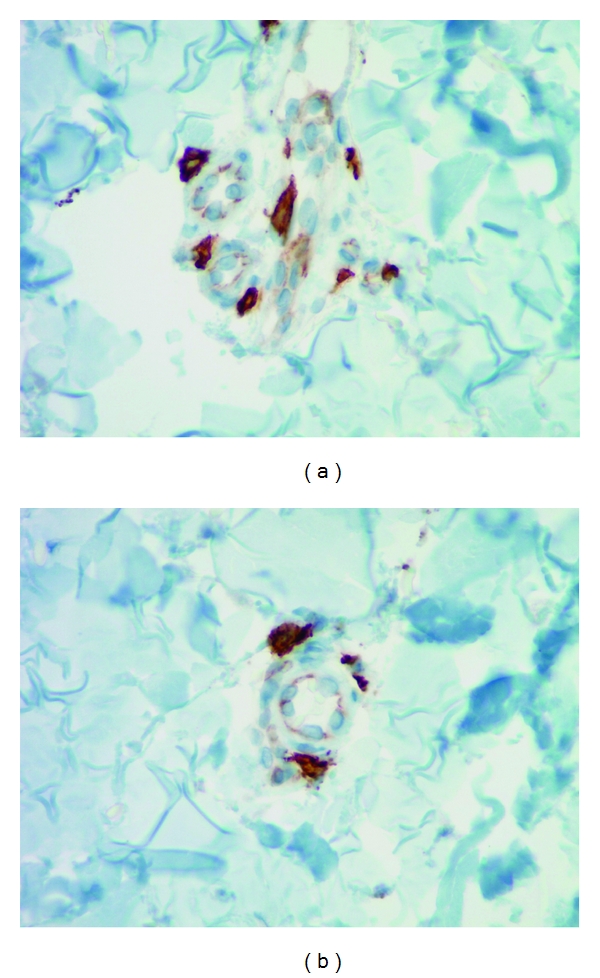
C-Kit (CD117) staining reveals the dendritic features of mast cells 40x.

**Figure 6:**
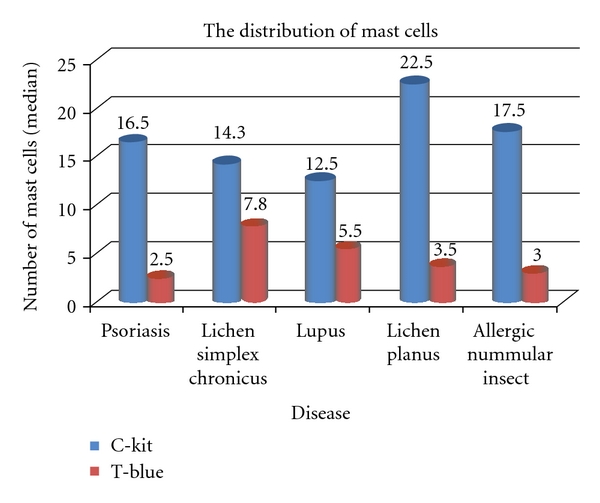


Our study revealed that the mast cell distribution pattern is different across the two methods of staining (CD-117 (C-kit) and toluidine blue) for the five aforesaid dermatoses. CD-117 (C-kit) staining in psoriasis, lupus, lichen planus, and insect bite/allergic contact dermatitis/nummular dermatitis is statistically significant in revealing the mast cells in skin biopsies compared to toluidine blue. The other important observation was the dendritic nature of the mast cells (Figures 5(a), 5(b) and 6). Some cells have only slender processes, whereas other cells have several long processes extending from the cell body. Some of these processes divide into two or three terminal branches. Our observation contributes new concepts to the heterogeneity of mast cells. Hence, human mast cells may vary with respect to morphologic features such as presence of dendritic processes as in our observation apart from the mediator contents.

In our understanding, this dendritic morphology may be due to their common origin from hematopoietic stem cell. The impact of mast cells on dendritic cell maturation and function has been studied by Dudeck et al. [12]. It was observed that mast cells primed dendritic cells stimulated CD4+ T cells to release high levels of cytokines, interferon-*γ* (IFN-*γ*), and interleukin-17 (IL-17). The dendritic cell migration, maturation, and cytokine release are affected by the histamine and tumor necrosis factor (TNF) released from mast cells following an IgE-dependent stimulation.

In addition to their role in immunity, mast cells participate also in the pathogenesis of fibrotic diseases and are found to stimulate fibroblast proliferation and collagen synthesis through some fibrotic mediators such as histamine and tryptase [13]. Studies have revealed that in presence of degranulated mast cells there was increased synthesis of type a1(I) procollagen mRNA. Mast cell tryptase stimulated fibroblast chemotaxis and also stimulated collagen mRNA synthesis [14]. Other functions of mast cells have been postulated in the sites of tumor formation around the squamous cells carcinomas, basal cell carcinomas, and angiosarcomas [3]. This may be of interest as activated mast cells can produce growth factors and growth and differentiation modulating factors such as IFN-*γ*, TNF, and vascular endothelial growth factor (VEGF).

The mast cell-targeting therapeutic approaches are under development following the identification of specific receptors on mast cells and elucidation of molecular mechanisms underlying activation of these cells [15]. These studies reveal a varied role of mast cell and their diagnostic and therapeutic importance in different diseases.

## 6. Conclusion

The role of mast cells as the “immune amplifiers” and the fact that activated mast cells can produce a broad range of immune mediators (such as IFN-*γ*, TNF, VEGF, endothelial growth factor (EGF), heparin, histamine, and matrix metalloproteinase) are of great interest for clarifying the task of mast cells in different diseases, for which quantification of mast cells is one of the initial steps in this pathway. Our study highlights a significant variation between the CD-117 (C-kit) and toluidine blue staining pattern for mast cells. Also the dendritic morphology of mast cells can further be studied using mast cell-specific stains like the chymase and tryptase. Hence-further studies need to be done using mast cell-specific immunohistochemical stains in order to quantify them and further understand their morphology and function in relation to their mediator contents.

## Figures and Tables

**Table 1:** Participant characteristics by disease. There were 24 people in the study (15 females and 9 males).

Characteristic	Psoriasis	Lichen simplex chronicus	Lupus	Lichen planus	Allergic/ nummular/insect	Total
Age, mean ± SD	37.2 ± 11.8	48.3 ± 10.9	47.2 ± 15.5	51.6 ± 13.6	19 ± 11.2	46.6 ± 12.7
Gender						
Female	3	2	5	2	3	15 (62.5%)
Male	2	2	0	3	2	9 (37.5%)

**Table 2:** Mast cells. It shows the outcome variable—the number of mast cells. Overall, the distribution of mast cells is different across the two methods (*P* < .0001). The median number of mast cells revealed using C-Kit method was 17.5 compared to the median number of mast cells revealed using T-Blue method. The distribution of mast cells is same across the two methods for lichen simplex chronicus (*P* = 0.110).

Characteristic	Median (p25, p75)^1^	*P* value^2^
Group		
C-Kit	17.5 (11.8; 22.5)	
T-Blue	3.8 (2.5; 7.0)	<.0001*

Psoriasis		
C-Kit	16.5 (16.0; 20.5)	
T-Blue	2.5 (1.0; 4.0)	0.012*

Lichen simplex chronicus		
C-Kit	14.3 (10.0; 35.0)	
T-Blue	7.8 (6.0; 12.8)	0.110

Lupus		
C-Kit	12.5 (6.0; 17.5)	
T-Blue	5.5 (3.5; 6.0)	0.024*

Lichen planus		
C-Kit	22.5 (20.0; 22.5)	
T-Blue	3.5 (0.5; 7.5)	0.036*

Allergic/nummular/insect		
C-Kit	17.5 (15.0; 22.5)	
T-Blue	3.0 (2.5; 3.0)	0.009*

^1^p25 represents the 25th percentile; p75 represents the 75th percentile.

^2^Mann-Whitney *U* test.

*indicates significance at 0.05 level of significance.
